# Trastuzumab-Induced Pneumonitis: A Rare but Potentially Severe Pulmonary Complication

**DOI:** 10.7759/cureus.105281

**Published:** 2026-03-15

**Authors:** Marco Antonio Rodríguez Sánchez, Ana Karen Verdiales Lugo, José Manuel Aguilar Rubio, Carlos Andres Olguin Guizado, Juliana Patricia Ortiz Jiménez, Carlos Orlando López Millán, Lucero Valenzuela Carvajal

**Affiliations:** 1 Department of Internal Medicine, Center for Research and Teaching in Health Sciences, Autonomous University of Sinaloa, Civil Hospital of Culiacán, Culiacán, MEX

**Keywords:** acute respiratory distress syndrome, corticosteroid therapy, ground-glass opacities, her2-positive breast cancer, trastuzumab-induced pneumonitis

## Abstract

Trastuzumab is a monoclonal antibody targeting the human epidermal growth factor receptor 2 (HER2) and is widely used in the treatment of HER2-positive breast cancer. Although generally well tolerated, trastuzumab has been rarely associated with pulmonary toxicity, including interstitial pneumonitis, a potentially life-threatening complication that may progress to acute respiratory distress syndrome (ARDS).

We report the case of a 75-year-old woman with HER2-positive pleomorphic lobular breast carcinoma treated with docetaxel, carboplatin, trastuzumab, and pertuzumab. Shortly after her fifth chemotherapy cycle, she developed acute respiratory failure characterized by severe hypoxemia. Chest computed tomography revealed bilateral ground-glass opacities with interstitial thickening and pleural effusions. Extensive infectious workup, including respiratory viral panels and bronchial cultures, was negative. Given the temporal relationship with trastuzumab exposure and exclusion of alternative diagnoses, trastuzumab-induced pneumonitis was suspected. The patient progressed to severe ARDS requiring invasive mechanical ventilation and prone positioning. High-dose systemic corticosteroid therapy resulted in significant initial clinical and gasometric improvement. However, her course was complicated by ventilator-associated pneumonia due to *Pseudomonas aeruginosa*, leading to a fatal outcome.

This case highlights the importance of early recognition of trastuzumab-induced pneumonitis, prompt exclusion of infectious and cardiogenic causes, and timely initiation of corticosteroid therapy to improve outcomes. Increased awareness of this rare but serious adverse event is essential as the use of anti-HER2 therapies continues to expand.

## Introduction

Trastuzumab is a monoclonal antibody targeting the human epidermal growth factor receptor 2 (HER2) and has significantly improved survival outcomes in patients with HER2-positive breast cancer. Although its safety profile is generally favorable, rare but serious adverse effects have been described, including pulmonary toxicity in the form of interstitial pneumonitis [1]. The reported incidence of trastuzumab-associated pneumonitis is low, estimated at approximately 0.5-1% in clinical trials, but severe cases may lead to life-threatening respiratory failure or even death [2].

Trastuzumab-induced pneumonitis typically presents with nonspecific respiratory symptoms such as dyspnea, cough, and hypoxemia, accompanied by radiological findings including bilateral ground-glass opacities and interstitial infiltrates [3]. These clinical and imaging features frequently mimic infectious pneumonia, cardiogenic pulmonary edema, or tumor progression, making early recognition challenging. Consequently, the diagnosis is usually established after exclusion of other potential causes of acute respiratory deterioration [4].

Evidence regarding this complication remains limited, and most available data derive from isolated case reports and small case series describing variable clinical presentations and outcomes. Reported cases have occurred in different clinical contexts, including patients receiving combination chemotherapy or targeted anti-HER2 therapy, with symptom onset typically occurring after several treatment cycles [5]. Given the increasing use of HER2-targeted therapies in oncology practice, awareness of this rare but potentially severe adverse event is essential for timely diagnosis and management.

## Case presentation

A 75-year-old woman with a six-month history of HER2-positive pleomorphic lobular breast carcinoma (histological grade 3) was referred to our institution. She had received neoadjuvant chemotherapy with docetaxel, carboplatin, trastuzumab, and pertuzumab (TCHP) every three weeks, completing five cycles. One month prior to admission, she underwent an uncomplicated right radical mastectomy.

Seven days after the fifth chemotherapy cycle, the patient developed progressive dyspnea, respiratory distress, and arterial oxygen desaturation. She was initially treated as an outpatient with empirical antibiotics and bronchodilator nebulizations. Despite this, her condition worsened, with the development of a productive cough, severe dyspnea, and oxygen saturation declining to 70%, prompting hospital admission.

On arrival, she was tachycardic (120 beats/min), tachypneic (30 breaths/min), afebrile, and markedly hypoxemic, with an oxygen saturation of 78% on room air. Laboratory evaluation revealed mild normocytic normochromic anemia (hemoglobin 11.1 g/dL), with normal leukocyte and platelet counts. Inflammatory markers, including C-reactive protein and procalcitonin, were negative. Arterial blood gas analysis demonstrated primary respiratory acidosis with severe hypoxemia (pH 7.30, pCO_2_ 59 mmHg, pO_2_ 27 mmHg). Pro-B-type natriuretic peptide (pro-BNP) levels were low (35 pg/mL), arguing against cardiogenic pulmonary edema (Table 1).

**Table 1:** Findings on admission. Reference ranges may vary according to institutional laboratory standards.

Parameter	Result	Reference range
Heart rate	120 bpm	60-100 bpm
Respiratory rate	30 breaths/min	12-20 breaths/min
Oxygen saturation (room air)	78%	≥94%
Hemoglobin	11.1 g/dL	12.0-16.0 g/dL
C-reactive protein	Negative	<5 mg/L
Procalcitonin	Negative	<0.05 ng/mL
Pro-B-type natriuretic peptide (pro-BNP)	35 pg/mL	<300 pg/mL
Arterial pH	7.30	7.35-7.45
Arterial pCO_2_	59 mmHg	35-45 mmHg
Arterial pO_2_	27 mmHg	80-100 mmHg

**Figure 1:**
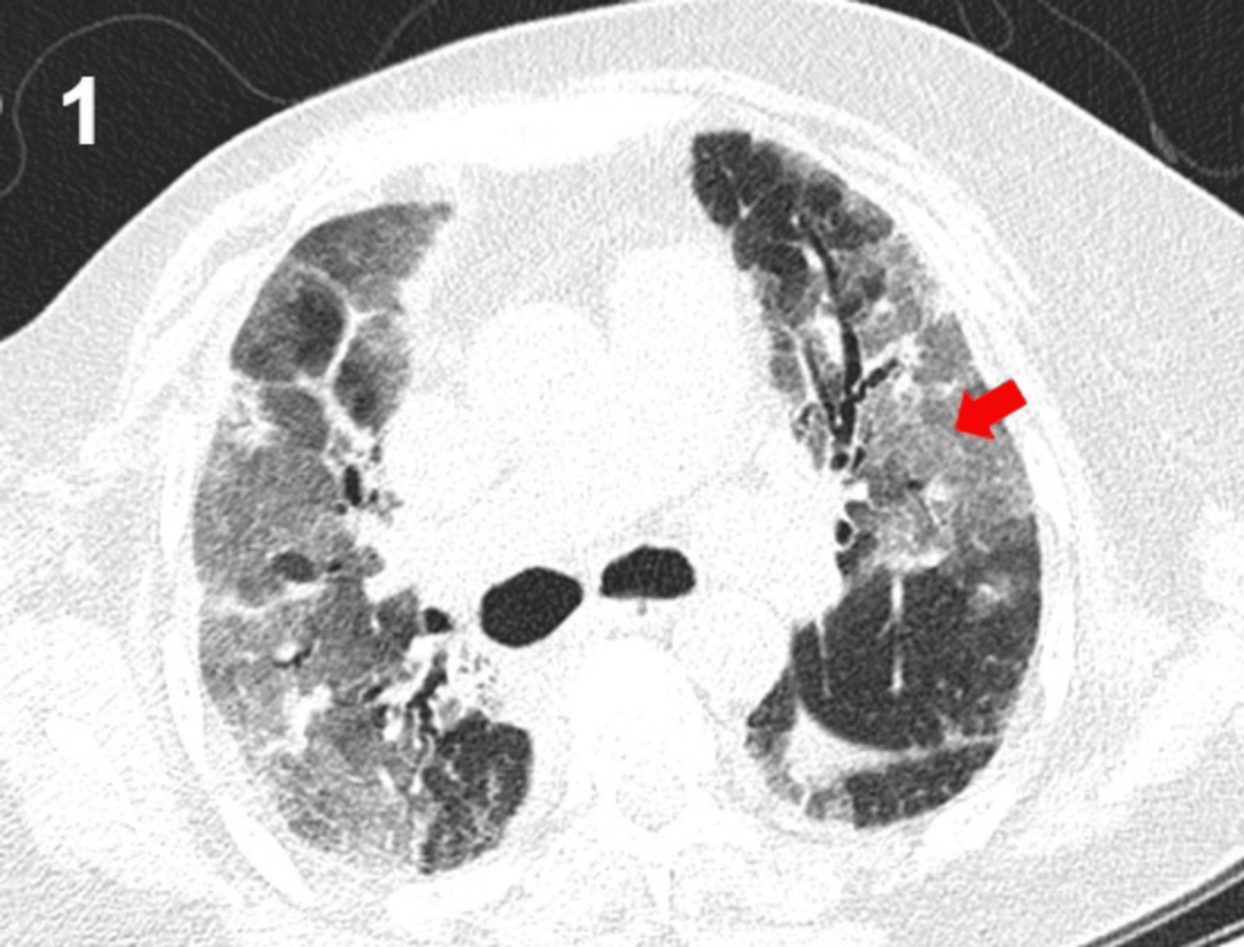
Computed tomography (CT) scan of the chest showing bilateral ground-glass opacities (red arrows) associated with intralobular and interlobular septal thickening, along with bilateral pleural effusions.

**Figure 2:**
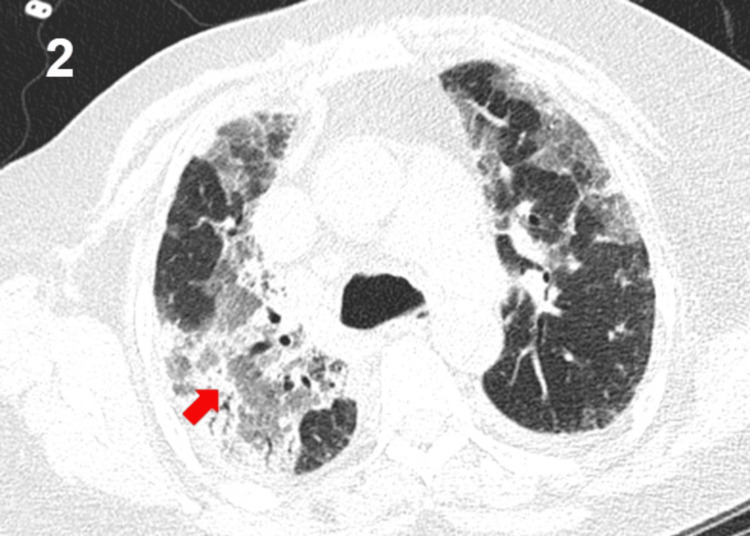
Computed tomography (CT) scan of the chest showing diffuse bilateral involvement with extensive ground-glass opacities and septal thickening, reflecting a generalized interstitial-alveolar pattern (red arrow). The extent and distribution of these findings correlate with severe hypoxemic respiratory failure and are characteristic of drug-induced pneumonitis in the appropriate clinical context.

The chest CT scan revealed bilateral ground-glass opacities with interlobular and alveolar septal thickening, along with bilateral pleural effusions (Figures 1-2), consistent with a diffuse interstitial-alveolar process. A respiratory viral panel, including influenza A and B, respiratory syncytial virus (RSV), SARS-CoV-2, adenovirus, parainfluenza viruses, rhinovirus/enterovirus, and human metapneumovirus, was performed and yielded negative results. Empirical treatment was initiated with oseltamivir, bronchodilators, and thromboprophylaxis. However, the patient's respiratory status deteriorated rapidly, with an increased need for oxygen. Despite the presence of tachycardia and tachypnea on admission, there were no clinical or laboratory findings suggestive of systemic infection. The patient was afebrile, and inflammatory markers, including C-reactive protein and procalcitonin, were negative, and the initial microbiological evaluation did not identify an infectious source. Therefore, a non-infectious etiology for the respiratory failure was considered more likely.

Given the temporal relationship with trastuzumab exposure, exclusion of infectious and cardiogenic causes, and compatible radiological findings, trastuzumab-induced pneumonitis was strongly suspected. The patient progressed to severe hypoxemic respiratory failure, with a PaO_2_/FiO_2_ ratio of 70, meeting criteria for severe acute respiratory distress syndrome (ARDS). She required endotracheal intubation, invasive mechanical ventilation, and early prone positioning.

High-dose intravenous methylprednisolone was initiated, leading to marked clinical and gasometric improvement, with gradual reduction in ventilatory support and improvement of the PaO_2_/FiO_2_ ratio to 195.

On the fifth day of intensive care unit (ICU) stay, the patient developed fever, increased bronchial secretions, and signs of systemic inflammatory response. Bronchial cultures isolated *Pseudomonas aeruginosa*, consistent with ventilator-associated pneumonia. Targeted antimicrobial therapy was promptly initiated according to the antibiogram. Despite appropriate management, the patient experienced progressive clinical deterioration and ultimately died on hospital day 15.

Clinical significance

This case highlights a rare but potentially fatal pulmonary complication associated with trastuzumab, emphasizing the importance of early recognition, exclusion of infectious causes, and prompt initiation of corticosteroid therapy. Furthermore, it underscores the vulnerability of critically ill oncology patients to secondary infectious complications, which may significantly impact outcomes.

## Discussion

Trastuzumab-induced pneumonitis is an uncommon but serious adverse event associated with anti-HER2 therapy, and most of the available evidence derives from isolated case reports and small case series reported in the literature (Table 2) [1-5]. Although the estimated incidence is less than 1%, morbidity and mortality are substantial, particularly in cases progressing to ARDS [2]. Our case illustrates the fulminant course that this complication may take, even in the absence of pre-existing pulmonary disease.

**Table 2:** Reported cases of trastuzumab-associated pneumonitis. GGO: ground-glass opacities; MV: mechanical ventilation; TCHP: docetaxel, carboplatin, trastuzumab, and pertuzumab

Author	Year	Age	Regimen	Onset after therapy	Imaging findings	Treatment	Outcome
Pepels et al. [4]	2009	59	Trastuzumab	Four months	Ground-glass opacities	Steroids	Recovery
Costa et al. [2]	2017	67	Trastuzumab + chemo	Three cycles	Interstitial infiltrates	Steroids	Recovery
Fontes et al. [6]	2022	65	Trastuzumab	Two months	Bilateral GGO	Steroids	Fatal
Present case	2026	75	TCHP	Five cycles	Bilateral GGO	Steroids + MV	Fatal

The pathophysiology remains incompletely understood, but proposed mechanisms include immune-mediated inflammation, diffuse alveolar damage, and impaired epithelial repair related to HER2 inhibition in pulmonary tissue. Several risk factors have been suggested, including combination therapy with taxanes, recent surgery, or prior radiotherapy [3]. In this case, previous exposure to docetaxel and recent mastectomy may have contributed to pulmonary vulnerability.

Clinically, the presentation is nonspecific and closely resembles infectious pneumonia or cardiogenic pulmonary edema [6]. In our patient, extensive microbiological testing was repeatedly negative, and low pro-BNP levels made cardiac causes unlikely. The temporal association with trastuzumab administration and characteristic imaging findings supported the diagnosis of drug-induced pneumonitis.

Although the patient received combination therapy including TCHP, several factors supported trastuzumab as the most likely causative agent of pneumonitis [1]. While docetaxel has been associated with drug-induced pneumonitis [7], pulmonary toxicity related to carboplatin is uncommon [8], and pneumonitis associated with pertuzumab appears to be rare in the literature [9].

Radiologically, bilateral ground-glass opacities with interstitial thickening are the most commonly reported findings in trastuzumab-related lung injury, consistent with patterns of interstitial pneumonitis or organizing pneumonia described in the literature. However, no pathognomonic imaging features exist, reinforcing that the diagnosis is largely one of exclusion [6].

Management recommendations are primarily extrapolated from reported cases and expert opinion. Immediate discontinuation of trastuzumab and early initiation of systemic corticosteroids constitute the cornerstone of treatment, particularly in moderate to severe cases [2,6]. Our patient demonstrated a clear initial response to high-dose intravenous methylprednisolone, with significant improvement in oxygenation and ventilatory requirements, supporting the inflammatory nature of the lung injury.

Despite this initial response, the patient subsequently developed ventilator-associated pneumonia caused by *P. aeruginosa*, a well-recognized complication in critically ill oncology patients requiring prolonged mechanical ventilation. This highlights an important clinical consideration: although corticosteroids may be life-saving in drug-induced pneumonitis, they may also increase susceptibility to secondary infections, which can ultimately influence prognosis.

## Conclusions

In conclusion, this case highlights a rare but potentially life-threatening pulmonary complication associated with anti-HER2 therapy. Although establishing definitive causality in complex oncologic patients can be challenging, the temporal relationship with trastuzumab exposure, the exclusion of infectious and cardiogenic etiologies, and the patient’s initial response to corticosteroid therapy supported a probable diagnosis of drug-induced pneumonitis.

This report underscores the importance of maintaining a high index of suspicion for therapy-related pulmonary toxicity in patients receiving HER2-targeted treatments who develop acute respiratory symptoms. Early recognition, prompt discontinuation of the suspected agent, and timely initiation of corticosteroid therapy may be critical to improving clinical outcomes. As the use of targeted oncologic therapies continues to expand, greater awareness of rare but severe adverse pulmonary events is essential to facilitate early diagnosis and appropriate management.
